# 4-[(*E*)-2-(4-{(*E*)-2-[4-((*E*)-2-{4-[(*E*)-2-(4-Cyanophenyl)ethen­yl]phenyl}ethen­yl)-2,5-bis(octyl­oxy)phenyl]ethen­yl}phenyl)ethen­yl]benzo­nitrile

**DOI:** 10.1107/S2414314626009120

**Published:** 2026-09-18

**Authors:** Heiner Detert, Dieter Schollmeyer

**Affiliations:** a University of Mainz, Department of Chemistry, Duesbergweg 10-14, 55099 Mainz, Germany; University of Aberdeen, United Kingdom

**Keywords:** crystal structure, phenyl­ene vinyl­ene, stilbene, oligomer

## Abstract

The complete mol­ecule of the title compound, C_56_H_60_N_2_O_2_, which was prepared *via* a Horner olefination, is generated by a crystallographic centre of symmetry. Whereas the terminal cyano­stilbene units are almost planar, the central dioctoxybenzene unit is substanti­ally tilted [dihedral angle = 51.68 (8)°], which limits conjugation. van der Waals forces between the alkyl chains connect the mol­ecules, arranged in layers lying parallel to the *ac* plane.

## Abstract 2

The complete mol­ecule of the title compound, which was prepared *via* a Horner olefination, is generated by a crystallographic centre of symmetry. Whereas the terminal cyano­stilbene units are almost planar, the central dioctoxybenzene unit is substanti­ally tilted, which limits conjugation. van der Waals forces between the alkyl chains connect the mol­ecules, arranged in layers lying parallel to the *ac* plane.

## Graphical abstract

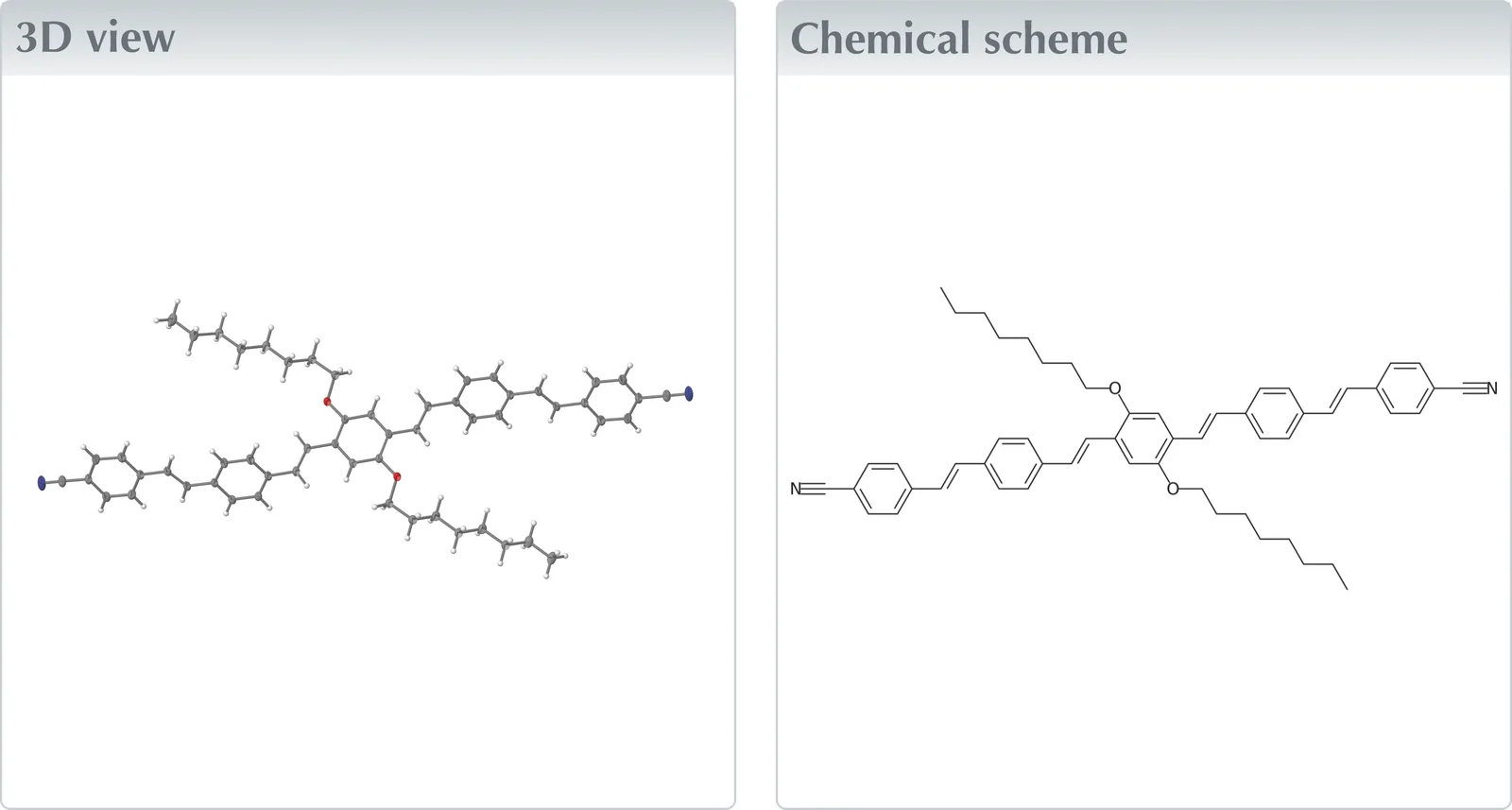

## Structure description

The title compound, C_56_H_60_N_2_O_2_ (**I**), is a linear *para*-oligo­phenyl­ene-vinyl­ene (OPV). It was prepared as part of a project on organic fluoro­phors with increased electron affinity (Detert & Sugiono, 2000 ▸; Stalmach & Detert, 2000 ▸; Detert & Schmitt, 2004 ▸). The structure of three related cyano-oligo-phenyl­ene­vinyl­enes with five benzene rings have been reported earlier (Gill *et al.* 1996 ▸; Detert *et al.* 2001 ▸) and the crystal structure of the unsubstituted 5-ring OPV has also been reported (van Hutten *et al.* 1999 ▸). Two mol­ecules of (**I**) fill the monoclinic unit cell. The centrosymmetric mol­ecules are composed of two terminal and almost planar stilbene units (ring 1 and 2) connected to a twisted di­vinyl­benzene moiety (ring 3) (Fig. 1 ▸). The terminal cyano groups and central oct­yloxy chains result formally in an electronic donor–acceptor–donor (*DAD*) structure. The key torsion angles of the stilbene unit are C9—C12—C13—C14 = −178.79 (15)°, C8—C9—C12—C13 = 179.36 (16)° and C19—C14—C13—C12 = 178.27 (16)° and the dihedral angle between the C6–C12 and the C14–C19 aromatic rings is 6.03 (8)°. Ring 2 (C6–C11) and the vinyl­ene unit subtend a torsion angle C7—C6—C5—C4 of −151.99 (17)° and the torsion angle between this vinyl­ene unit and the central ring 3 (C5—C4—C3—C1^i^) [symmetry code: (i) 1 − *x*, 1 − *y*, 1–*z*) amounts to 156.00 (16)°. The dihedral angle between the central ring and the C6–C12 ring is 51.68 (8)°. Whereas the first methyl­ene groups show a *gauche* conformation about the C21—C22 bond [O20—C21—C22—C23 = 60.83 (19)°], the rest of the octyl chain is perfectly *all-anti* configured.

In the extended structure of (**I**), the mol­ecules are arranged in alternating layers lying parallel to the *ac* plane (Fig. 2 ▸). The rings 1 and 2 are almost parallel to the *ac*-plane whereas the central ring is tilted: the dihedral angle between the central rings 3 and 3′ of the alternating layers amounts to 71.50 (7)°. The alkyl chains connect parallel layers of mol­ecules, along the *b* axis, one chain inter­acts with the chains of mol­ecules above and one with those below, forming aliphatic layers between the conjugated segments, also lying roughly parallel to the *ac* plane.

## Synthesis and crystallization

A solution of potassium-*t*-butyl­ate (336 mg, 3 mmol) and 60 mg 18-crown-6 in 30 ml of anhydrous tetra­hydro­furan (THF) was purged with nitro­gen and a solution of 594 mg (1 mmol) 1,4-bis­(4-formyl­phenyl­ethen­yl)-2,5-dioctyloxyben­zene (Detert *et al.* 2001 ▸). 2 mmol (506 mg) *p*-cyano­benzyl phospho­nate in 15 ml of anhydrous THF was added dropwise over 15 min. Stirring was continued for 16 h before the reaction mixture was poured into 150 ml of 0.5 N hydro­chloric acid. The product was isolated by filtration, the residue was washed with 100 ml of water/methanol (2/1) dried in vacuum and recrystallized from chloro­form solution by adding methanol to the hot solution in 0.66 g (83%) yield. Recrystallization for single crystals was from the mixed solvents of chloro­form and 2-propanol. Bright yellow crystals with m.p. = 481 K. MS (FD): 793 (100) [*M*^+^], 396.5 (1) [*M*^2+^]; IR (KBr): ν = 3010, 2908, 2840, 2220, 1590, 1582, 1502, 1480, 1453, 1408, 1248, 1195, 1019, 957, 850, 830, 820 cm^−1^; ^1^H-NMR (CDCl_3_, 400 MHz): δ = 0.88 (*t*, *J* = 6.4 Hz, 6 H, CH_3_), 1.25–1.45 (*m*, 20 H, CH_2_), 1.56 (*qui*, *J* = 7.5 Hz, 4 H, CH_2_), 1.87 (*qui*, *J* = 7.5 Hz, 4 H, CH_2_), 4.07 (*t*, *J* = 6 Hz, 4 H, OCH_2_), 7.06 (*d*, *J* = 16.4 Hz, 2 H, vin), 7.11 (*s*, 2 H), 7.13 (*d*, *J* = 16 Hz, 2 H, vin), 7.19 (*d*, *J* = 16.4 Hz, 2 H, vin), 7.53 (*d*, *J* = 16 Hz, 2 H, vin), 7.52 (*AA*’*BB*’, 8 H), 7.59 (*AA*’*BB*’, 8 H); ^13^C-NMR (CDCl_3_, 100 MHz): δ = 14.1 (CH_3_), 22.7, 26.3, 29.3, 29.4, 29.5, 31.8 (CH_2_), 69.6 (OCH_2_), 110.5, 110.7, 119.0 (CN), 124.1, 126.4 (CH vin), 126.8, 127.0, 127.3 (CH ar), 138.3, 132.0 (CH vin), 132.5 (CH ar), 135.4, 138.5, 141.9, 151.3 (Cq). UV-Vis l max = 434 nm (di­chloro­methane, log ɛ = 4.93), fluorescence: λ_max_ = 485 nm (cyclo­hexane, Φ = 0.53), λ_max_ = 521 (aceto­nitrile).

## Refinement

Crystal data, data collection and structure refinement details are summarized in Table 1 ▸.

## Supplementary Material

Crystal structure: contains datablock(s) I, global. DOI: 10.1107/S2414314626009120/hb4571sup1.cif

Structure factors: contains datablock(s) I. DOI: 10.1107/S2414314626009120/hb4571Isup2.hkl

Supporting information file. DOI: 10.1107/S2414314626009120/hb4571Isup3.cml

CCDC reference: 2584677

Additional supporting information:  crystallographic information; 3D view; checkCIF report

## Figures and Tables

**Figure 1:**
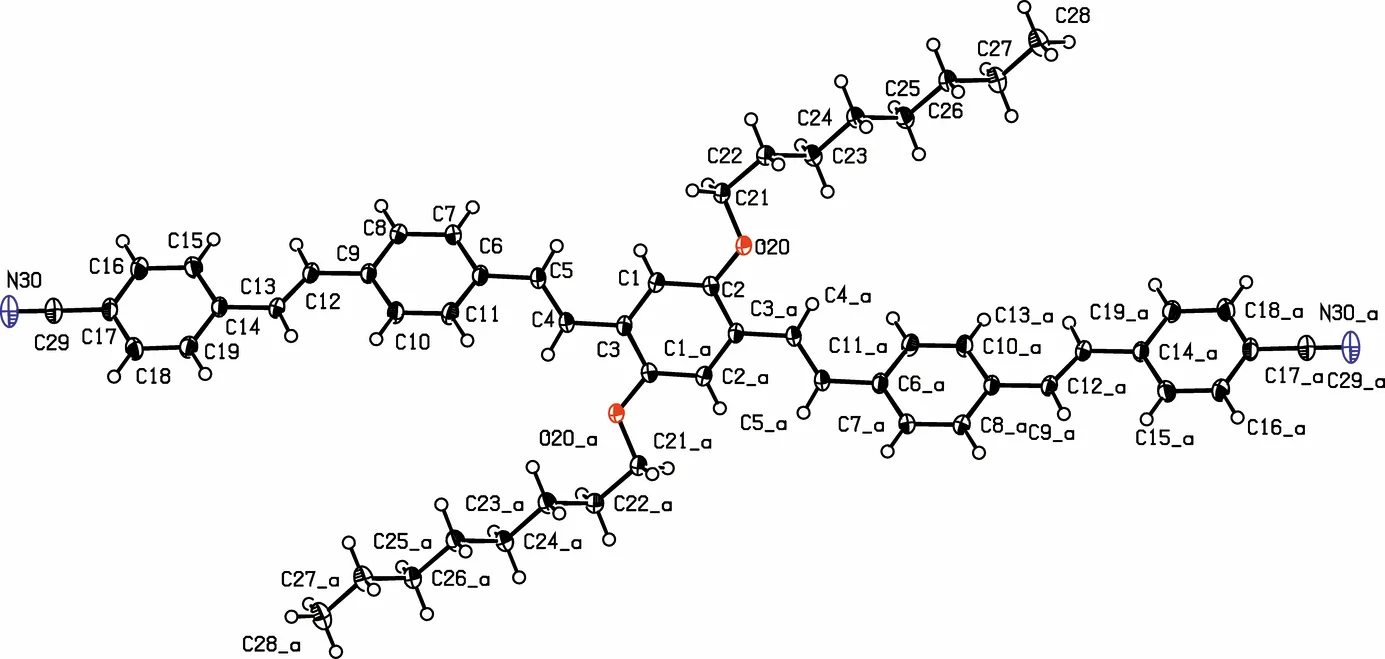
The mol­ecular structure of (**I**) with displacement ellipsoids drawn at the 50% probability level. Symmetry code: (a) 1 − *x*, 1 − *y*, 1 − *z*.

**Figure 2:**
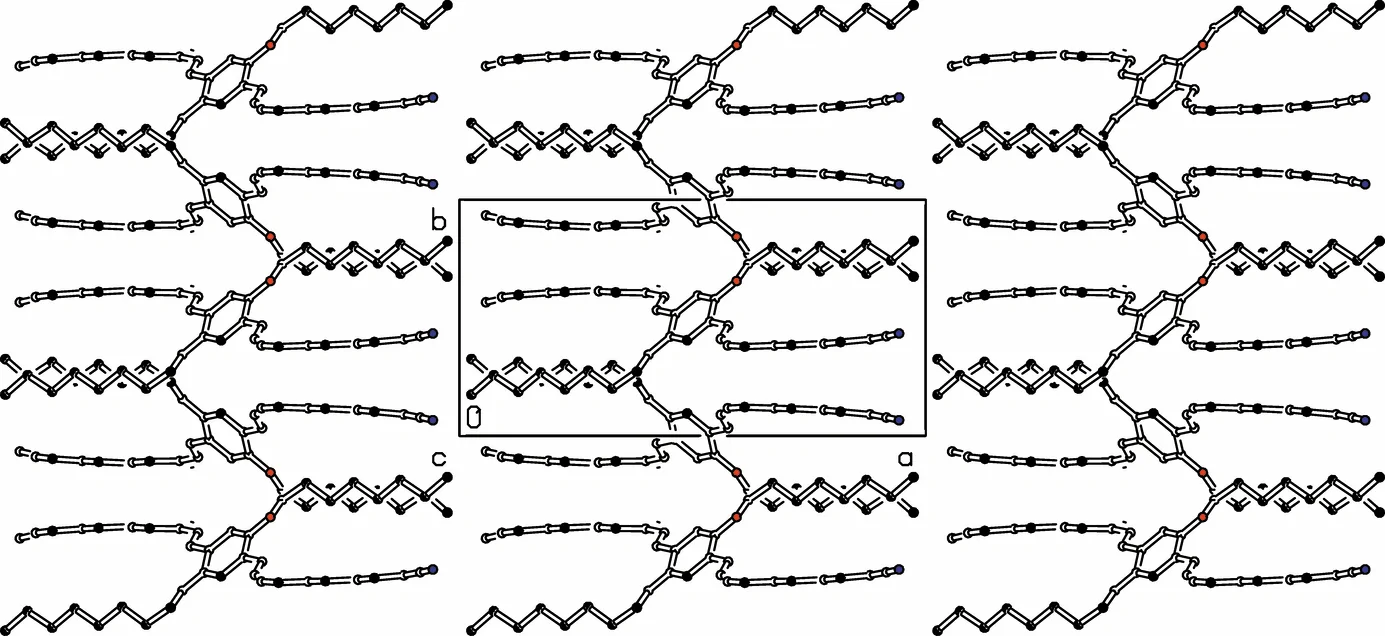
Part of the packing diagram of (**I**) viewed along the *c*-axis direction. Hydrogen atoms omitted for clarity.

**Table 1:** Experimental details

Crystal data	
Chemical formula	C_56_H_60_N_2_O_2_
*M*_r_	793.06
Crystal system, space group	Monoclinic, *P*2_1_/*c*
Temperature (K)	120
*a*, *b*, *c* (Å)	22.0613 (7), 11.1029 (3), 9.1280 (3)
β (°)	96.004 (3)
*V* (Å^3^)	2223.59 (12)
*Z*	2
Radiation type	Mo *K*α
μ (mm^−1^)	0.07
Crystal size (mm)	0.26 × 0.15 × 0.05

Data collection	
Diffractometer	Stoe *IPDS* 2T
Absorption correction	–
No. of measured, independent and observed [*I* > 2σ(*I*)] reflections	14627, 5254, 3801
*R*_int_	0.060
(sin θ/λ)_max_ (Å^−1^)	0.658

Refinement	
*R*[*F*^2^ > 2σ(*F*^2^)], *wR*(*F*^2^), *S*	0.056, 0.150, 1.10
No. of reflections	5254
No. of parameters	272
H-atom treatment	H-atom parameters constrained
Δρ_max_, Δρ_min_ (e Å^−3^)	0.32, −0.23

Computer programs: *X-AREA WinXpose*, *X-AREA Recipe* and *X-AREA Integrate* (Stoe & Cie, 2020 ▸), *SHELXT2014* (Sheldrick, 2015*a* ▸), *SHELXL2019/2* (Sheldrick, 2015*b* ▸) and *PLATON* (Spek, 2009 ▸).

## full crystallographic data

### 4-[(E)-2-(4-{(E)-2-[4-((E)-2-{4-[(E)-2-(4-Cyanophenyl)ethenyl]phenyl}ethenyl)-2,5-bis(octyloxy)phenyl]ethenyl}phenyl)ethenyl]benzonitrile Crystal data

**Table:**

C_56_H_60_N_2_O_2_	*F*(000) = 852
*M**_r_* = 793.06	*D*_x_ = 1.184 Mg m^−^^3^
Monoclinic, *P*2_1_/*c*	Mo *K*α radiation, λ = 0.71073 Å
*a* = 22.0613 (7) Å	Cell parameters from 10876 reflections
*b* = 11.1029 (3) Å	θ = 2.9–28.4°
*c* = 9.1280 (3) Å	µ = 0.07 mm^−^^1^
β = 96.004 (3)°	*T* = 120 K
*V* = 2223.59 (12) Å^3^	Plate, yellow
*Z* = 2	0.26 × 0.15 × 0.05 mm

### 4-[(E)-2-(4-{(E)-2-[4-((E)-2-{4-[(E)-2-(4-Cyanophenyl)ethenyl]phenyl}ethenyl)-2,5-bis(octyloxy)phenyl]ethenyl}phenyl)ethenyl]benzonitrile Data collection

**Table:**

Stoe IPDS 2T diffractometer	*R*_int_ = 0.060
Detector resolution: 6.67 pixels mm^-1^	θ_max_ = 27.9°, θ_min_ = 2.9°
rotation method, ω scans	*h* = −28→28
14627 measured reflections	*k* = −14→14
5254 independent reflections	*l* = −11→10
3801 reflections with *I* > 2σ(*I*)	

### 4-[(E)-2-(4-{(E)-2-[4-((E)-2-{4-[(E)-2-(4-Cyanophenyl)ethenyl]phenyl}ethenyl)-2,5-bis(octyloxy)phenyl]ethenyl}phenyl)ethenyl]benzonitrile Refinement

**Table:**

Refinement on *F*^2^	Primary atom site location: dual
Least-squares matrix: full	Hydrogen site location: inferred from neighbouring sites
*R*[*F*^2^ > 2σ(*F*^2^)] = 0.056	H-atom parameters constrained
*wR*(*F*^2^) = 0.150	*w* = 1/[σ^2^(*F*_o_^2^) + (0.0575*P*)^2^ + 0.8682*P*] where *P* = (*F*_o_^2^ + 2*F*_c_^2^)/3
*S* = 1.10	(Δ/σ)_max_ = 0.001
5254 reflections	Δρ_max_ = 0.32 e Å^−^^3^
272 parameters	Δρ_min_ = −0.23 e Å^−^^3^
0 restraints	

### 4-[(E)-2-(4-{(E)-2-[4-((E)-2-{4-[(E)-2-(4-Cyanophenyl)ethenyl]phenyl}ethenyl)-2,5-bis(octyloxy)phenyl]ethenyl}phenyl)ethenyl]benzonitrile Special details

**Table:**

Geometry. All esds (except the esd in the dihedral angle between two l.s. planes) are estimated using the full covariance matrix. The cell esds are taken into account individually in the estimation of esds in distances, angles and torsion angles; correlations between esds in cell parameters are only used when they are defined by crystal symmetry. An approximate (isotropic) treatment of cell esds is used for estimating esds involving l.s. planes.
Refinement. Hydrogen atoms attached to carbon atoms were placed at calculated positions and were refined in the riding-model approximation with C—H = 0.95–0.99 Å, and with *U*_iso_(H) = 1.2 *U*_eq_(C).

### 4-[(E)-2-(4-{(E)-2-[4-((E)-2-{4-[(E)-2-(4-Cyanophenyl)ethenyl]phenyl}ethenyl)-2,5-bis(octyloxy)phenyl]ethenyl}phenyl)ethenyl]benzonitrile Fractional atomic coordinates and isotropic or equivalent isotropic displacement parameters (Å^2^)

**Table:**

	*x*	*y*	*z*	*U*_iso_*/*U*_eq_	
C1	0.54781 (7)	0.57938 (15)	0.53929 (17)	0.0239 (3)	
C2	0.51135 (7)	0.59457 (15)	0.40744 (17)	0.0250 (3)	
H2	0.519388	0.659476	0.344480	0.030*	
C3	0.46279 (7)	0.51606 (14)	0.36499 (17)	0.0231 (3)	
C4	0.42353 (7)	0.53272 (14)	0.22687 (17)	0.0234 (3)	
H4	0.383510	0.500323	0.221817	0.028*	
C5	0.43916 (7)	0.58960 (15)	0.10723 (17)	0.0243 (3)	
H5	0.479187	0.621900	0.111866	0.029*	
C6	0.39939 (7)	0.60616 (14)	−0.03081 (17)	0.0227 (3)	
C7	0.42449 (7)	0.61702 (15)	−0.16410 (18)	0.0253 (3)	
H7	0.467510	0.621205	−0.163772	0.030*	
C8	0.38796 (7)	0.62187 (15)	−0.29725 (17)	0.0249 (3)	
H8	0.406367	0.628526	−0.386451	0.030*	
C9	0.32444 (7)	0.61709 (13)	−0.30210 (16)	0.0212 (3)	
C10	0.29919 (7)	0.61295 (15)	−0.16739 (17)	0.0253 (3)	
H10	0.256141	0.613619	−0.167190	0.030*	
C11	0.33561 (7)	0.60793 (15)	−0.03533 (17)	0.0255 (3)	
H11	0.317221	0.605628	0.054207	0.031*	
C12	0.28738 (7)	0.61536 (14)	−0.44523 (17)	0.0227 (3)	
H12	0.308816	0.618492	−0.530154	0.027*	
C13	0.22659 (7)	0.60985 (14)	−0.46871 (16)	0.0232 (3)	
H13	0.204763	0.608294	−0.384361	0.028*	
C14	0.19060 (7)	0.60598 (14)	−0.61326 (17)	0.0222 (3)	
C15	0.21729 (8)	0.60958 (17)	−0.74565 (18)	0.0305 (4)	
H15	0.260282	0.617067	−0.743152	0.037*	
C16	0.18224 (8)	0.60242 (17)	−0.87991 (18)	0.0314 (4)	
H16	0.201187	0.605442	−0.968637	0.038*	
C17	0.11929 (7)	0.59082 (15)	−0.88546 (18)	0.0257 (3)	
C18	0.09147 (7)	0.58874 (16)	−0.75575 (19)	0.0296 (4)	
H18	0.048438	0.581748	−0.758965	0.036*	
C19	0.12712 (7)	0.59699 (16)	−0.62128 (18)	0.0282 (4)	
H19	0.107929	0.596498	−0.532842	0.034*	
O20	0.59453 (5)	0.65600 (11)	0.58728 (12)	0.0294 (3)	
C21	0.61954 (8)	0.72790 (17)	0.47691 (19)	0.0312 (4)	
H21A	0.588076	0.782783	0.429117	0.037*	
H21B	0.634359	0.675574	0.400389	0.037*	
C22	0.67179 (8)	0.79956 (16)	0.55458 (19)	0.0297 (4)	
H22A	0.655559	0.851893	0.629142	0.036*	
H22B	0.688589	0.852632	0.481586	0.036*	
C23	0.72361 (7)	0.72408 (15)	0.63057 (19)	0.0280 (4)	
H23A	0.741249	0.673462	0.556451	0.034*	
H23B	0.707294	0.669872	0.703115	0.034*	
C24	0.77349 (7)	0.80304 (16)	0.70892 (18)	0.0275 (4)	
H24A	0.790550	0.854940	0.635149	0.033*	
H24B	0.755105	0.856283	0.779200	0.033*	
C25	0.82516 (7)	0.73201 (15)	0.79204 (19)	0.0276 (4)	
H25A	0.844567	0.680762	0.721451	0.033*	
H25B	0.808084	0.678287	0.863937	0.033*	
C26	0.87357 (7)	0.81255 (15)	0.87348 (18)	0.0271 (3)	
H26A	0.889982	0.867307	0.801669	0.033*	
H26B	0.854171	0.862822	0.945071	0.033*	
C27	0.92614 (8)	0.74288 (17)	0.9551 (2)	0.0321 (4)	
H27A	0.909921	0.688541	1.027633	0.039*	
H27B	0.945598	0.692386	0.883831	0.039*	
C28	0.97400 (8)	0.82510 (19)	1.0348 (2)	0.0376 (4)	
H28A	0.989332	0.880827	0.964013	0.056*	
H28B	1.007783	0.776397	1.081085	0.056*	
H28C	0.955712	0.871022	1.110644	0.056*	
C29	0.08398 (8)	0.57799 (16)	−1.02704 (19)	0.0302 (4)	
N30	0.05706 (8)	0.56627 (16)	−1.14068 (18)	0.0424 (4)	

### 4-[(E)-2-(4-{(E)-2-[4-((E)-2-{4-[(E)-2-(4-Cyanophenyl)ethenyl]phenyl}ethenyl)-2,5-bis(octyloxy)phenyl]ethenyl}phenyl)ethenyl]benzonitrile Atomic displacement parameters (Å^2^)

**Table:**

	*U*^11^	*U*^22^	*U*^33^	*U*^12^	*U*^13^	*U*^23^
C1	0.0224 (7)	0.0294 (8)	0.0190 (7)	−0.0037 (6)	−0.0026 (6)	0.0007 (6)
C2	0.0266 (8)	0.0299 (8)	0.0175 (7)	−0.0036 (6)	−0.0029 (6)	0.0040 (6)
C3	0.0227 (7)	0.0296 (8)	0.0161 (7)	−0.0010 (6)	−0.0023 (6)	0.0017 (6)
C4	0.0223 (7)	0.0277 (8)	0.0189 (7)	−0.0030 (6)	−0.0048 (6)	0.0006 (6)
C5	0.0238 (7)	0.0286 (8)	0.0188 (7)	−0.0017 (6)	−0.0047 (6)	0.0003 (6)
C6	0.0266 (8)	0.0228 (7)	0.0173 (7)	−0.0009 (6)	−0.0038 (6)	0.0010 (6)
C7	0.0213 (7)	0.0323 (8)	0.0212 (8)	−0.0025 (6)	−0.0034 (6)	0.0036 (6)
C8	0.0255 (8)	0.0307 (8)	0.0181 (7)	−0.0019 (6)	−0.0001 (6)	0.0023 (6)
C9	0.0243 (7)	0.0212 (7)	0.0170 (7)	−0.0002 (6)	−0.0030 (6)	0.0005 (6)
C10	0.0219 (7)	0.0345 (9)	0.0186 (7)	0.0039 (6)	−0.0014 (6)	0.0017 (6)
C11	0.0256 (8)	0.0338 (9)	0.0166 (7)	0.0039 (6)	0.0006 (6)	0.0000 (6)
C12	0.0271 (8)	0.0247 (7)	0.0157 (7)	0.0003 (6)	−0.0013 (6)	0.0007 (6)
C13	0.0261 (8)	0.0280 (8)	0.0149 (7)	−0.0003 (6)	−0.0010 (6)	0.0006 (6)
C14	0.0240 (7)	0.0237 (7)	0.0178 (7)	−0.0002 (6)	−0.0032 (6)	0.0011 (6)
C15	0.0233 (8)	0.0467 (10)	0.0210 (8)	−0.0053 (7)	−0.0002 (6)	−0.0013 (7)
C16	0.0306 (9)	0.0463 (10)	0.0168 (8)	−0.0042 (8)	−0.0001 (6)	0.0001 (7)
C17	0.0283 (8)	0.0266 (8)	0.0202 (8)	0.0004 (6)	−0.0071 (6)	0.0009 (6)
C18	0.0212 (7)	0.0400 (9)	0.0264 (8)	0.0013 (7)	−0.0037 (6)	0.0022 (7)
C19	0.0255 (8)	0.0381 (9)	0.0204 (8)	0.0015 (7)	0.0006 (6)	0.0017 (7)
O20	0.0315 (6)	0.0366 (7)	0.0183 (5)	−0.0139 (5)	−0.0067 (5)	0.0048 (5)
C21	0.0318 (9)	0.0371 (9)	0.0224 (8)	−0.0114 (7)	−0.0071 (7)	0.0078 (7)
C22	0.0305 (8)	0.0323 (9)	0.0250 (8)	−0.0084 (7)	−0.0037 (7)	0.0055 (7)
C23	0.0272 (8)	0.0297 (8)	0.0257 (8)	−0.0050 (7)	−0.0035 (7)	0.0001 (7)
C24	0.0268 (8)	0.0308 (8)	0.0236 (8)	−0.0057 (7)	−0.0034 (6)	−0.0006 (7)
C25	0.0255 (8)	0.0300 (8)	0.0262 (8)	−0.0021 (7)	−0.0019 (6)	−0.0018 (7)
C26	0.0265 (8)	0.0304 (8)	0.0234 (8)	−0.0025 (7)	−0.0025 (6)	−0.0009 (7)
C27	0.0272 (8)	0.0358 (9)	0.0316 (9)	0.0034 (7)	−0.0046 (7)	−0.0047 (7)
C28	0.0276 (8)	0.0458 (11)	0.0373 (10)	0.0022 (8)	−0.0061 (7)	−0.0069 (8)
C29	0.0296 (8)	0.0333 (9)	0.0257 (9)	0.0041 (7)	−0.0066 (7)	−0.0008 (7)
N30	0.0448 (9)	0.0499 (10)	0.0288 (8)	0.0090 (8)	−0.0138 (7)	−0.0058 (7)

### 4-[(E)-2-(4-{(E)-2-[4-((E)-2-{4-[(E)-2-(4-Cyanophenyl)ethenyl]phenyl}ethenyl)-2,5-bis(octyloxy)phenyl]ethenyl}phenyl)ethenyl]benzonitrile Geometric parameters (Å, º)

**Table:**

C1—O20	1.3726 (18)	C17—C18	1.389 (2)
C1—C2	1.386 (2)	C17—C29	1.445 (2)
C1—C3^i^	1.408 (2)	C18—C19	1.390 (2)
C2—C3	1.404 (2)	C18—H18	0.9500
C2—H2	0.9500	C19—H19	0.9500
C3—C4	1.465 (2)	O20—C21	1.4398 (19)
C4—C5	1.337 (2)	C21—C22	1.514 (2)
C4—H4	0.9500	C21—H21A	0.9900
C5—C6	1.470 (2)	C21—H21B	0.9900
C5—H5	0.9500	C22—C23	1.525 (2)
C6—C7	1.394 (2)	C22—H22A	0.9900
C6—C11	1.404 (2)	C22—H22B	0.9900
C7—C8	1.387 (2)	C23—C24	1.525 (2)
C7—H7	0.9500	C23—H23A	0.9900
C8—C9	1.398 (2)	C23—H23B	0.9900
C8—H8	0.9500	C24—C25	1.522 (2)
C9—C10	1.403 (2)	C24—H24A	0.9900
C9—C12	1.467 (2)	C24—H24B	0.9900
C10—C11	1.378 (2)	C25—C26	1.525 (2)
C10—H10	0.9500	C25—H25A	0.9900
C11—H11	0.9500	C25—H25B	0.9900
C12—C13	1.337 (2)	C26—C27	1.522 (2)
C12—H12	0.9500	C26—H26A	0.9900
C13—C14	1.468 (2)	C26—H26B	0.9900
C13—H13	0.9500	C27—C28	1.522 (2)
C14—C19	1.398 (2)	C27—H27A	0.9900
C14—C15	1.399 (2)	C27—H27B	0.9900
C15—C16	1.381 (2)	C28—H28A	0.9800
C15—H15	0.9500	C28—H28B	0.9800
C16—C17	1.391 (2)	C28—H28C	0.9800
C16—H16	0.9500	C29—N30	1.148 (2)
			
O20—C1—C2	123.36 (14)	C18—C19—C14	121.47 (15)
O20—C1—C3^i^	115.85 (13)	C18—C19—H19	119.3
C2—C1—C3^i^	120.76 (14)	C14—C19—H19	119.3
C1—C2—C3	121.39 (15)	C1—O20—C21	116.76 (12)
C1—C2—H2	119.3	O20—C21—C22	107.00 (13)
C3—C2—H2	119.3	O20—C21—H21A	110.3
C2—C3—C1^i^	117.86 (14)	C22—C21—H21A	110.3
C2—C3—C4	121.64 (14)	O20—C21—H21B	110.3
C1^i^—C3—C4	120.50 (14)	C22—C21—H21B	110.3
C5—C4—C3	125.64 (14)	H21A—C21—H21B	108.6
C5—C4—H4	117.2	C21—C22—C23	114.96 (15)
C3—C4—H4	117.2	C21—C22—H22A	108.5
C4—C5—C6	125.26 (14)	C23—C22—H22A	108.5
C4—C5—H5	117.4	C21—C22—H22B	108.5
C6—C5—H5	117.4	C23—C22—H22B	108.5
C7—C6—C11	117.50 (14)	H22A—C22—H22B	107.5
C7—C6—C5	120.22 (14)	C22—C23—C24	111.55 (14)
C11—C6—C5	122.25 (14)	C22—C23—H23A	109.3
C8—C7—C6	121.38 (15)	C24—C23—H23A	109.3
C8—C7—H7	119.3	C22—C23—H23B	109.3
C6—C7—H7	119.3	C24—C23—H23B	109.3
C7—C8—C9	120.97 (15)	H23A—C23—H23B	108.0
C7—C8—H8	119.5	C25—C24—C23	113.70 (14)
C9—C8—H8	119.5	C25—C24—H24A	108.8
C8—C9—C10	117.51 (14)	C23—C24—H24A	108.8
C8—C9—C12	119.47 (14)	C25—C24—H24B	108.8
C10—C9—C12	123.02 (14)	C23—C24—H24B	108.8
C11—C10—C9	121.30 (15)	H24A—C24—H24B	107.7
C11—C10—H10	119.3	C24—C25—C26	112.89 (14)
C9—C10—H10	119.3	C24—C25—H25A	109.0
C10—C11—C6	121.14 (15)	C26—C25—H25A	109.0
C10—C11—H11	119.4	C24—C25—H25B	109.0
C6—C11—H11	119.4	C26—C25—H25B	109.0
C13—C12—C9	126.83 (14)	H25A—C25—H25B	107.8
C13—C12—H12	116.6	C27—C26—C25	113.53 (15)
C9—C12—H12	116.6	C27—C26—H26A	108.9
C12—C13—C14	125.77 (14)	C25—C26—H26A	108.9
C12—C13—H13	117.1	C27—C26—H26B	108.9
C14—C13—H13	117.1	C25—C26—H26B	108.9
C19—C14—C15	117.81 (14)	H26A—C26—H26B	107.7
C19—C14—C13	119.59 (14)	C28—C27—C26	112.59 (16)
C15—C14—C13	122.60 (14)	C28—C27—H27A	109.1
C16—C15—C14	121.17 (15)	C26—C27—H27A	109.1
C16—C15—H15	119.4	C28—C27—H27B	109.1
C14—C15—H15	119.4	C26—C27—H27B	109.1
C15—C16—C17	120.13 (15)	H27A—C27—H27B	107.8
C15—C16—H16	119.9	C27—C28—H28A	109.5
C17—C16—H16	119.9	C27—C28—H28B	109.5
C18—C17—C16	119.93 (15)	H28A—C28—H28B	109.5
C18—C17—C29	121.06 (15)	C27—C28—H28C	109.5
C16—C17—C29	118.99 (15)	H28A—C28—H28C	109.5
C17—C18—C19	119.47 (15)	H28B—C28—H28C	109.5
C17—C18—H18	120.3	N30—C29—C17	178.4 (2)
C19—C18—H18	120.3		
			
O20—C1—C2—C3	−177.93 (15)	C12—C13—C14—C19	178.27 (16)
C3^i^—C1—C2—C3	0.1 (3)	C12—C13—C14—C15	−1.0 (3)
C1—C2—C3—C1^i^	−0.1 (3)	C19—C14—C15—C16	−1.1 (3)
C1—C2—C3—C4	179.06 (15)	C13—C14—C15—C16	178.19 (17)
C2—C3—C4—C5	24.8 (3)	C14—C15—C16—C17	−0.3 (3)
C1^i^—C3—C4—C5	−156.00 (16)	C15—C16—C17—C18	1.2 (3)
C3—C4—C5—C6	−179.83 (15)	C15—C16—C17—C29	−177.20 (16)
C4—C5—C6—C7	−151.99 (17)	C16—C17—C18—C19	−0.7 (3)
C4—C5—C6—C11	26.0 (3)	C29—C17—C18—C19	177.69 (16)
C11—C6—C7—C8	−3.9 (2)	C17—C18—C19—C14	−0.7 (3)
C5—C6—C7—C8	174.14 (15)	C15—C14—C19—C18	1.6 (3)
C6—C7—C8—C9	0.6 (3)	C13—C14—C19—C18	−177.68 (15)
C7—C8—C9—C10	2.9 (2)	C2—C1—O20—C21	−21.7 (2)
C7—C8—C9—C12	−176.47 (15)	C3^i^—C1—O20—C21	160.09 (15)
C8—C9—C10—C11	−3.0 (2)	C1—O20—C21—C22	−177.28 (14)
C12—C9—C10—C11	176.31 (15)	O20—C21—C22—C23	60.83 (19)
C9—C10—C11—C6	−0.3 (3)	C21—C22—C23—C24	−178.65 (14)
C7—C6—C11—C10	3.8 (2)	C22—C23—C24—C25	177.60 (14)
C5—C6—C11—C10	−174.23 (15)	C23—C24—C25—C26	−178.22 (14)
C8—C9—C12—C13	179.35 (16)	C24—C25—C26—C27	−179.01 (14)
C10—C9—C12—C13	0.0 (3)	C25—C26—C27—C28	179.70 (15)
C9—C12—C13—C14	−178.79 (15)		

Symmetry code: (i) −*x*+1, −*y*+1, −*z*+1.
